# Harnessing the Role of *ESR1* in Breast Cancer: Correlation with microRNA, lncRNA, and Methylation

**DOI:** 10.3390/ijms26073101

**Published:** 2025-03-27

**Authors:** Shengping Yang, Chayan Manna, Pulak R. Manna

**Affiliations:** 1 Pennington Biomedical Research Center, 6400 Perkins Rd., Baton Rouge, LA 70808, USA; 2 Baylor College of Medicine, Ben Taub Research Center, 1 Baylor Plaza, Houston, TX 77030, USA; cmanna.bcm-medical@pm.me; 3 Department of Internal Medicine, School of Medicine, Texas Tech University Health Sciences Center, 3601 4th Street, Lubbock, TX 79430, USA

**Keywords:** breast cancer, *ESR1*, gene expression, lncRNA, microRNA, methylation

## Abstract

Breast cancer (BC) is a multifactorial condition and it primarily expresses the estrogen receptor α (ERα) that is encoded by the gene estrogen receptor 1 (*ESR1*), which modulates estrogen signaling. *ESR1*, by facilitating estrogen overproduction, plays an indispensable role in the progression and survival of the majority of BCs. To obtain molecular insights into these phenomena, we analyzed The Cancer Genome Atlas (TCGA) breast invasive carcinoma (BRCA) RNA-Seq datasets for the expression of *ESR1* and its correlation to microRNAs (miRNAs) and long non-coding RNAs (lncRNAs), along with its methylation patterns. Regulation of *ESR1* was also assessed with a total of 43 cancerous and non-cancerous breast cell lines. Analyses of both TCGA BRCA and breast cell line RNA-Seq data revealed that specific lncRNAs, i.e., MEG3, BIK, MLL, and FAS are negatively correlated with the *ESR1*, in which PARP1 demonstrates a positive association. Additionally, both miR-30a and miR-145 showed negative correlations with the *ESR1* expression. Of the 54 *ESR1* methylation loci analyzed, the majority of them exhibited a negative correlation with the *ESR1* expression, highlighting a potentially modifiable regulatory mechanism. These findings underscore the complex regulatory events influencing *ESR1* expression and its interaction with diverse signaling pathways, demonstrating novel insights into breast pathogenesis and its potential therapeutics.

## 1. Introduction

Breast cancer (BC) is one of the most frequently diagnosed malignancies in women worldwide [1]. BC is the second leading cause of cancer-related death among women, with an estimated 316,950 new cases and 42,170 deaths occurring in 2025 in the United States [2]. BC is a multifactorial disease including four molecular subtypes, i.e., luminal A (ER+, PR+, and human epidermal growth factor receptor-negative, Her−), luminal B (ER+, PR+, and Her+), Her2 (ER−, PR−, and Her+), and basal like (ER−, PR−, and Her−, also called triple-negative BC, TNBC) [3,4,5]. Therefore, BC heterogeneity exhibits unique biological and clinical features, which considerably influence prognosis and treatment strategies [6,7]. For instance, hormone receptor-dependent BCs (≥80% of all cases) largely express ER, especially *ESR1*, progesterone receptor (PR+), and/or HER2+, all of which are responsible for the growth and maintenance of BCs [8,9]. Treatment typically involves endocrine therapy with aromatase inhibitors (AIs), which either block ER and/or reduce estrogen levels [9,10,11]. In contrast, triple-negative BC (TNBC, 10–15%) is hormone-independent and does not express ER, PR, or HER2; thus, it is unresponsive to endocrine therapy [12,13]. Regardless of the diverse factors and pathways involved, the activation of estrogen signaling, involving its overproduction, is a predominant event in breast pathogenesis [14,15,16].

The actions of estrogens are mediated by nuclear ERs, ERα and ERβ (encoded by *ESR1* and *ESR2* genes, respectively), which play pivotal roles in a plethora of physiological, as well as pathophysiological, processes that involve both genomic and non-genomic signaling [17,18,19]. Whereas both *ESR1* and *ESR2* display considerable sequence homology, they exert diverse actions in tumor initiation and development [6,19]. Notably, *ESR1* is activated by estrogens and it plays a key role in the growth and progression of hormone-sensitive BC. Mutations and/or variations in *ESR1* can affect how BC responds to endocrine therapy, making it a critical factor for the prognosis and treatment of this life-threatening disease [20,21,22,23]. Even so, BC heterogeneity, involving epidemiological patterns, differently influences *ESR1* regulation, necessitating an in-depth understanding of the mechanisms with diverse signaling [24,25,26].

Long non-coding RNAs (lncRNAs) constitute those that are longer than 200 nucleotides that do not code for proteins and have been involved in various malignant and non-malignant diseases [27,28]. LncRNAs regulate gene expression through various mechanisms, including chromatin remodeling, transcription, and post-transcriptional events [29,30]. Moreover, a number of lncRNAs have been shown to play crucial roles in the regulation of *ESR1* expression and activity, impacting breast pathogenesis [31,32,33].

MicroRNAs (miRNAs) are small non-coding RNAs (~22 nucleotides) that regulate gene expression by binding to complementary sequences, leading to either degradation or translational inhibition [34,35]. Concomitantly, both miR-30a and miR-145 have been implicated in the regulation of *ESR1* expression, and their interactions contribute to the progression and treatment of BCs. MicroRNAs have been reported to influence estrogen signaling, thus contributing to endocrine therapy for mitigating BCs [36,37].

Methylation of the *ESR1* promoter can silence its expression, resulting in a reduction in estrogen levels. Hypomethylation of *ESR1* is characteristically associated with ER+/PR+ BC, maintaining hormone responsiveness. Studies have shown that methylation of the *ESR1* promoter is considered a predictive marker in BC [38,39,40]. Moreover, environmental factors, such as diet, obesity, and exposure to endocrine-disrupting chemicals, alter *ESR1* methylation patterns. This suggests that *ESR1* methylation could be a modifiable factor influencing BC risk via modifications of genetic and epigenetic signaling [41].

An overwhelming amount of evidence indicates that TCGA RNA-Seq datasets offer a comprehensive understanding of the molecular basis of BC and other malignant disorders through high-throughput genome sequencing “https://gdc.cancer.gov/about-data/publications/pancanatlas (accessed on 25 January 2025)” [6,9,42,43,44,45]. To gain knowledge of the molecular events involved in breast pathogenesis, we analyzed both TCGA BRCA [42] and breast cell line [46] RNA-Seq data for the expression of *ESR1* and its correlation to miRNA, lncRNA, and methylation. Our data advance the field and provide novel insights into *ESR1* regulation and its interaction with a variety of signaling, permitting a better understanding of breast carcinogenesis, in addition to prospective therapeutics, especially for the ER+/PR+ BC subtype.

## 2. Results and Discussion

BC is characterized by aberrant and uncontrolled growth of mammary epithelial cells that involve genetic abnormality in modulating DNA damage and genomic instability [6,45,47]. Disruption of the equilibrium between oncogenes and tumor suppressor genes results in breast tumorigenesis, and this event is primarily impacted by the upregulation of estrogen signaling [48,49]. Utilizing TCGA BRCA datasets, we reported that genetic and epigenetic irregularities, impacting *ESR1* function, are common in human primary breast tumors that are mirrored in pertinent breast cell lines [6,9]. Notably, *ESR1* plays a crucial role in the pathogenesis of BCs and many relevant disorders. In accordance with this, we reported that amplification of the *ESR1* correlates with poor survival of BC patients [6]. Analyzing TCGA BRCA, as well as breast cell line RNA-Seq datasets, our current data extend these observations and elucidate an improved understanding of breast carcinogenesis by demonstrating the molecular insights into the interacting mechanisms between *ESR1*, lncRNA, and miRNA, along with methylation.

### 2.1. Expression of the ESR1 in the TCGA BRCA Datasets with Various BC Subtypes

**Figure 1:**
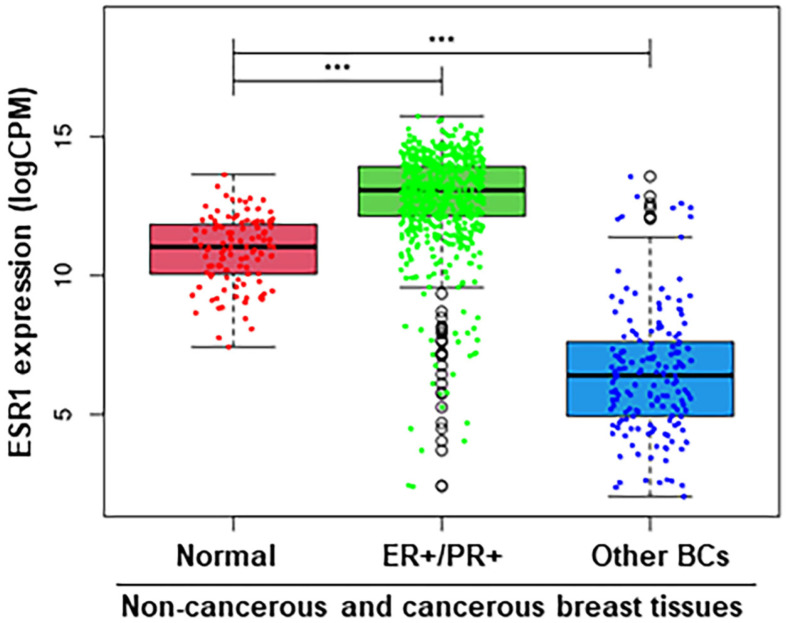
Boxplot analysis of the *ESR1* expression using TCGA BRCA RNA-Seq datasets under three different categories: Normal (114), ER+/PR+ (611), and Other BCs (164), with sample numbers in parentheses. ***, *p* < 0.001 vs. Normal, as indicated.

Since the activation of estrogen signaling, modulating estrogen synthesis is a primary event in breast tumorigenesis [16,50], TCGA BRCA (lrgely ER+/PR+ subtypes [45]) RNA-Seq datasets were examined for the relative expression of the *ESR1* in non-cancerous (normal) and cancerous (ER+/PR+ and other BCs) breast tissues. As shown by box plot analyses (Figure 1), ER+/PR+ BC samples showed substantially higher *ESR1* expression (*p* < 0.001), compared with both normal and other BC types, in which the latter includes hormone-independent/TNBC. Other BC subtypes displayed significantly lower expression of *ESR1* (*p* < 0.001) than that of non-cancerous normal breast tissue. These data indicate that *ESR1* expression, enhancing estrogen levels, plays an important role for in various BC subtypes in comparison to normal breast tissue.

### 2.2. Analyses of TCGA BRCA and Breast Cell Line RNA-Seq Datasets for a Number of lncRNAs and Their Correlation to ESR1

**Figure 2:**
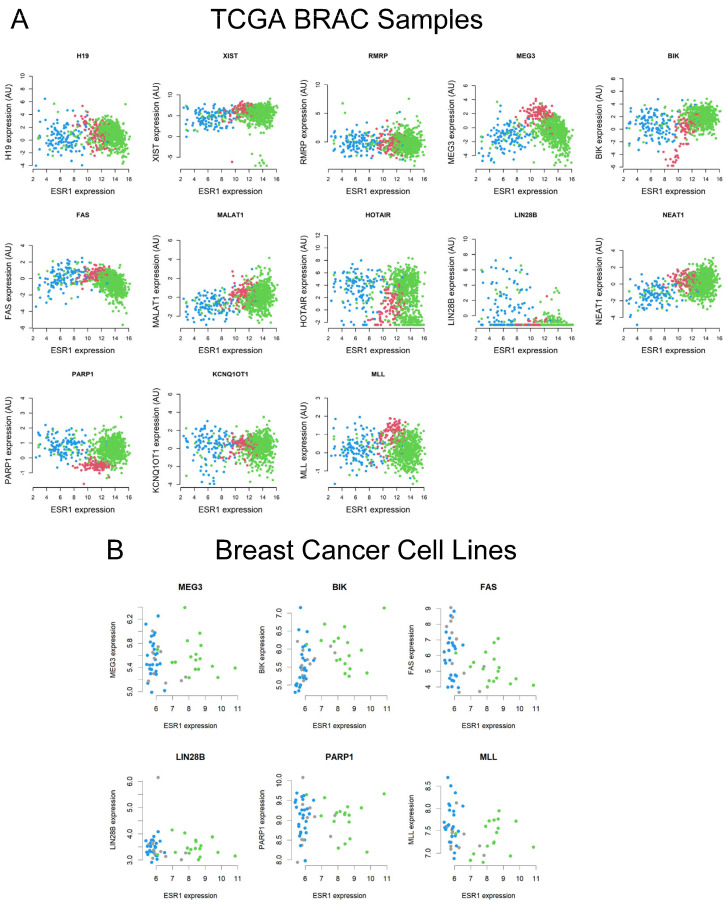
Analyses of the Spearman’s correlation coefficients between *ESR1* and specific lncRNAs (H19, XIST, RMRP, MEG3, BIK, FAS, MALAT1, HOTAIR, LIN28B, NEAT1, PARP1, KCNQ10T1, and MLL) using TCGA BRCA RNA-Seq data (**A**). The colors used were normal tissue—red; ER+/PR+ samples—green; and other BC subtypes—blue. (**B**) The Spearman’s correlation coefficients between *ESR1* and target lncRNAs (MEG3, BIK, FAS, LIN 28B, PARP1, and MLL) with 43 breast cell line RNA-Seq data. Colors used were ER+ samples—green; ER− samples—blue; and other subtypes—gray.

There is increasing evidence that expression of the *ESR1* is higher in ER+/PR+ BC, compared with normal breast tissue samples; however, hormone-independent BCs exhibit low to no *ESR1* levels [6,8,9]. We observed that a number of lncRNAs such as MEG3, MLL, and BIK show higher expression patterns in both hormone-sensitive and other BC subtypes (Figure 2). To investigate the molecular interaction between *ESR1* and target lncRNAs, TCGA BRCA and breast cell line RNA-Seq datasets were analyzed, converging normal tissue and ER+/PR+ BC. The pairwise comparison results revealed that the Spearman’s correlation coefficients between *ESR1* and lncRNAs, i.e., H19, MEG3, BIK, FAS, LIN28B, NEAT1, PARP1, and MLL were −0.20, −0.46, 0.28, −0.48, −0.16, 0.12, 0.26, and −0.19, respectively, with TCGA BRCA data. These correlations are significant at *p* < 0.001 for H19, MEG3, BIK, FAS, LIN28B, NEAT1, PARP1, and MLL. Alternatively, LIN28B is correlated at *p* = 0.018 (Figure 2A). Additionally, the Spearman’s correlation coefficients for MEG3, BIK, FAS, LIN28B, PARP1, and MLL were −0.06, 0.41, −0.42, −0.11, −0.02, and −0.26, respectively (Figure 2B), using a variety of breast cell line RNA-Seq data [46]. Among these lncRNAs, whereas BIK and FAS were positively correlated at *p* = 0.002 and *p* = 0.004, FAS showed a negative association (*p* = 0.004) with *ESR1*, respectively. The results obtained in breast cell lines are consistent with human primary breast cancer data associated with TCGA RBCA.

**Figure 3:**
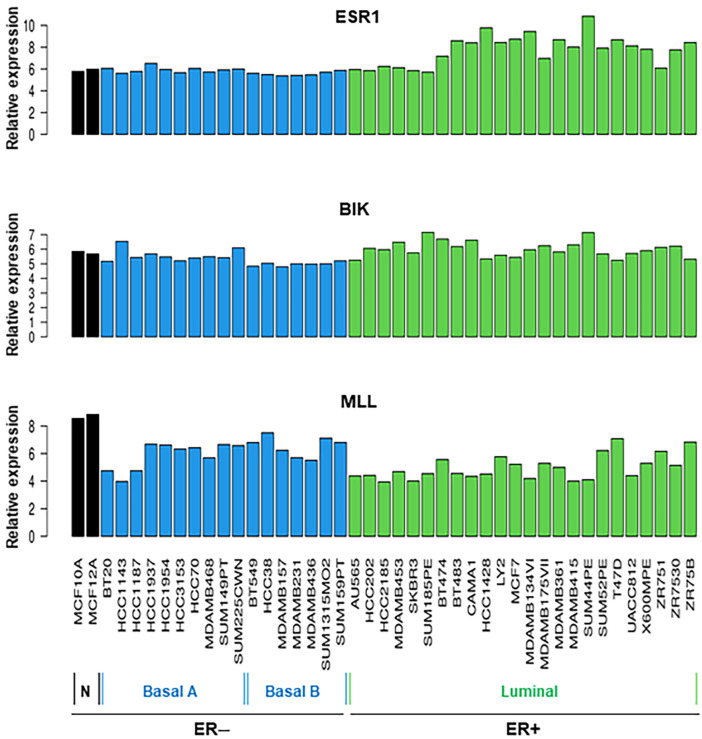
Analyses of RNA-Seq data for relative expression of *ESR1*, BIK, and MLL mRNAs in a total of 43 different cancerous and non-cancerous breast cell lines. Shown are the names of various breast cell lines under the ER− and ER+ categories.

Breast tissue cell line RNA-Seq datasets were further analyzed for the relative expression of *ESR1*, BIK, and MLL. Data presented in Figure 3 show that all 43 cancerous and non-cancerous cell lines express *ESR1* at varying levels, regardless of ER− and ER+ status. However, the expression of *ESR1* mRNA was higher in the ER+ category than in those ER− subtypes. Genomic profiles of BIK and MLL indicated that all breast cell lines analyzed express these lncRNAs to varied levels; however, the expression of BIK and MLL mRNA levels was found to be diverse in ER+ BC cell lines. These data suggest that *ESR1*, BIK, and MLL play important roles in breast physiology and pathophysiology.

Accumulating evidence indicates that upregulation of MEG3 inhibits the malignant features of cells in vitro [51,52,53]. Moreover, MEG3 acts as a tumor suppressor by epigenetically regulating target genes [54]. Even so, it has been shown that high MEG3 expression impacts the growth of breast cancer in vivo [55]. Studies have demonstrated that MEG3 exerts multifaceted function in various pathological processes, including BCs, by interacting with miRNA, proteins, and epigenetic signaling, and its relevance has been reported in many clinical applications [54,56,57]. Another lncRNA, MLL, modulates H3K4me3 at the *ESR1* promoter, maintaining open chromatin for active transcription. Loss or mutation of MLL has been shown to decrease *ESR1* expression, potentially contributing to reducing ER signaling in BCs [58,59]. Additionally, PARP1 plays an important role in regulating *ESR1* target genes. Enhanced PARP1 activity may promote hormone-driven cancer progression by impacting ER-mediated transcription. Co-inhibition of PARP1 and ER signaling has been implicated as a potential therapeutic strategy for BCs [60,61]. The inverse relationship between MEG3 and PARP1 designates that MEG3 could inhibit DNA repair mechanisms critical for cancer cell survival. This interaction might make MEG3 a valuable modulator in therapies targeting the PARP1 with inhibitors, particularly in tumors with defective apoptotic pathways. Genetic aberrations of *ESR1* and lncRNAs associated with human primary breast tumors in TCGA were closely reflected with various breast cell line data, highlighting an improved understanding of the mechanisms involved in breast tumorigenesis and its therapies. Estrogen signaling via *ESR1* upregulates the expression of several metabolic enzymes, including FAS, to support lipid metabolism in BC cells. In support of this, studies have shown that cholesterol and its oxygenated derivatives are drivers of BCs [6,62,63]. FAS converts acetyl-CoA and malonyl-CoA into palmitate and plays a critical role in providing lipid synthesis, energy storage, and signaling in rapidly BC and other malignant cells [64]. Moreover, estrogen can directly or indirectly regulate FAS expression, suggesting a link between hormone signaling and lipid metabolism [65,66,67]. It is likely that the interaction of *ESR1* with specific lncRNAs, but not their expression levels, plays an important role in breast pathogenesis and can be targeted for improved BC therapeutics.

### 2.3. Correlation of Specific miRNAs with ESR1 and Their Relevance to Breast Pathogenesis

**Figure 4:**
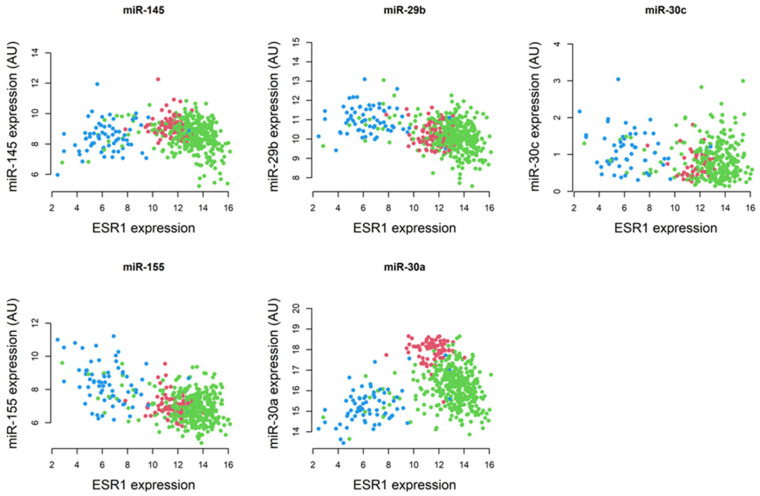
The Spearman’s correlation coefficients between *ESR1* and miRNAs (miR-145, miR-29b, miR-30c, miR-155, and miR-30a) using TCGA BRCA RNA-Seq data. Colors used were normal tissue—red; ER/PR+ samples—green; and other BC subtypes—blue.

MiRNAs post-transcriptionally regulate gene expression and, by doing so, they influence diverse cell signaling processes, including proliferation, differentiation, and apoptosis, and promote breast tumor progression [68,69]. Considering their involvement, miRNAs have been targeted in the diagnosis and treatment of BCs [70,71,72,73]. By analyzing TCGA BRCA RNA-Seq data, we found that the expression of miR-29b and miR-155 is lower in ER+/PR+ BC and higher in hormone-independent subtypes, compared with normal breast tissue. In contrast, miR-145 and miR-30a revealed higher expression in non-tumorous mammary tissue. Pairwise comparisons between *ESR1* and miRNAs in normal breast tissue and ER+/PR+ BCs revealed that the Spearman’s correlation coefficients between *ESR1* and miR-145, miR-29b, miR-155, and miR-30a were −0.30, −0.21, −0.17, and −0.36, respectively. The corresponding *p*-values for these correlations were significant at *p* < 0.001 for miR-145, miR-29b, and miR-30a, and *p* < 0.003 for miR-155 (Figure 4).

MiR-30a directly targets the 3′ UTR of *ESR1* mRNA, reducing its expression. This post-transcriptional repression has been reported to affect the ERα protein in BC cells [74]. Also, miR-145 is known to downregulate *ESR1* expression by binding to its mRNA, leading to its degradation or translational repression. Studies have demonstrated that miR-145 suppresses cell proliferation and invasion in BC, especially in TNBC [75,76]. The elevated levels of miR-30a in normal breast tissue contribute to cellular homeostasis by disrupting *ESR1,* which impacts apoptotic pathways. Conversely, its downregulation in ER+/PR+ BCs involves epigenetic silencing and/or transcriptional repression, emphasizing its loss for BC development and progression. Nonetheless, whereas a number of miRNAs, including miR-30a, have been implicated in therapeutic targets for BCs and other relevant diseases [77,78], their clinical applications have yet to be validated. Taken together, regardless of varied correlation patterns of selected lncRNAs and miRNAs with the *ESR1*, Kaplan–Meier survival curve analyses indicated that none of these lncRNAs and miRNAs were found to affect the overall survival of BC patients (Yang et al., unpublished observations).

### 2.4. Analyses of TCGA BRCA Data for ESR1 DNA Methylation Patterns

The majority of mammalian gene promoters are comprehended within regions of the genome called CpG islands, which are DNA regions with high CpG frequency and low methylation influencing oncogenic signaling and regulation [79,80,81]. We analyzed TCGA BRCA RNA-Seq data and found that a strong correlation exists between the DNA methylation of various CpG islands and the *ESR1* gene expression (Table 1). In particular, a significant negative correlation is observed between *ESR1* and the methylation of many CpG sites, such as cg00601836, cg15543523, and cg21265702, with correlation coefficients ranging from −0.26 to −0.68 and unadjusted *p*-values as low as 6.44 × 10^−58^. These findings indicate that higher methylation levels in these CpG sites are associated with lower expression of the *ESR1*. The adjusted *p*-values for these correlations remain significant even after multiple testing corrections. Interestingly, cg25490334 displayed a positive correlation with the *ESR1* expression (correlation = 0.21), with a higher *p*-value at 5.25 × 10^−6^. Overall, these data indicate that DNA methylation plays an important regulatory role in the expression of *ESR1*, with specific CpG sites showing strong inverse relationships with the *ESR1* activity. A detailed distribution pattern of DNA methylation and the *ESR1* expression is presented in Supplementary Figure S1.

### 2.5. Generation of a Heatmap Depicting a Correlation Matrix with lncRNAs, miRNAs, and ESR1

**Figure 5:**
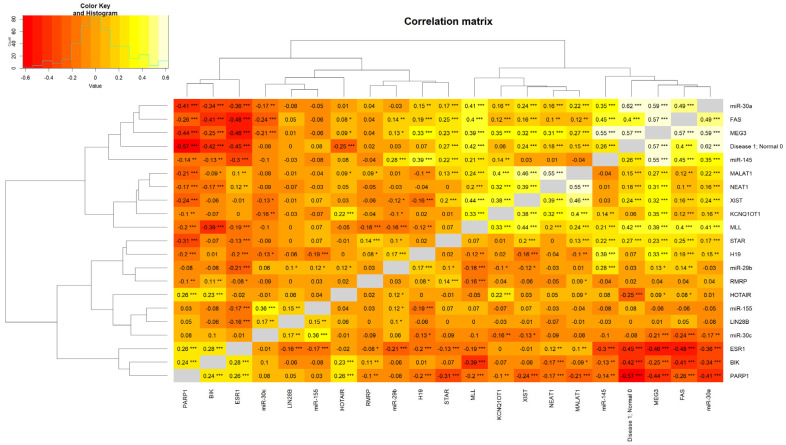
A heatmap illustrating the correlation matrix among lncRNA, miRNA, and *ESR1*. The genes included on the correlation map that influence diverse signaling pathways were the following: miR-30a, FAS, MEG3, miR-145, MALAT1, NEAT1, XIST, KCNQ10T1, MLL, STAR, H19, miR-29b, RMRP, HOTAIR, miR-155, LIN288, miR-30c, *ESR1*, BIK, and RARP1. *p*-values are provided in the correlation map in the format of “*” 0.01 ≤ *p* < 0.05; “**” 0.001 ≤ *p* < 0.01; and “***” *p* < 0.001.

An interesting aspect of the present findings is strong correlations among various lncRNAs and miRNAs with the *ESR1* (Figure 5). A number of lncRNAs and miRNAs, by interacting with *ESR1*, are known to activate estrogen signaling, triggering breast tumorigenesis [82,83,84,85]. Robust positive associations were observed between MEG3 and FAS (correlation = 0.57; *p* < 0.001), miR-30a (correlation = 0.59; *p* < 0.001), and miR-145 (correlation = 0.55; *p* < 0.001). Alternatively, negative correlations were found with PARP1 (correlation = −0.44; *p* < 0.001) and *ESR1* (correlation = −0.46; *p* < 0.001). Moreover, miR-30a is negatively correlated with *ESR1* and positively correlated with MLL (correlation = 0.41; *p* < 0.001) and MEG3 and FAS (correlation = 0.49; *p* < 0.001). In addition, the miR-30a level is much higher in normal breast tissue in comparison to ER+/PR+ BC (correlation = 0.62, *p* < 0.001). Overall, the relationships observed highlight the complexity of interactions in the regulation of various genes and their implications in normal physiologic, as well as pathophysiologic (hormone-responsive BC), states.

Correlation matrix data revealed the presence of a positive association between miR-30a and MLL, influencing chromatin remodeling and activation of tumor-suppressor genes, which reinforce the anti-tumorigenic effects of miR-30a in BC [86,87]. The tumor-suppressive role of MEG3 appears to be multifaceted, in which it is positively correlated with miR-30a, miR-145, and FAS in promoting apoptosis. These correlations suggest that MEG3 may serve as a platform for orchestrating regulatory networks and enhancing tumor suppression. The negative correlations with PARP1 and *ESR1* align with their tumor-suppressive role by diminishing pathways associated with DNA repair and hormone-driven proliferation, respectively. The negative correlations of MEG3 and miR-30a with *ESR1* emphasize their potential roles in reducing estrogen-driven proliferation. This inverse relationship supports therapeutic strategies aimed at modulating *ESR1* through MEG3 and miR-30a or targeting epigenetic modifications affecting *ESR1* expression. There is an interplay between miRNAs and lncRNAs in tumor suppression [88,89]. The positive correlations between MEG3 and miR-30a, as well as miR-145, specify cooperative mechanisms, in which MEG3 may act as a competing endogenous RNA, sequestering oncogenic miRNAs to allow tumor-suppressive function effectively. Co-regulation of these lncRNAs through common upstream pathways, such as the p53 signaling axis, provides further insights into their interdependence and highlights the therapeutic potential in BCs [90].

*ESR1* methylation, involving environmental and lifestyle factors, offers perceptions of the epigenetic plasticity of hormone receptor genes. This plasticity represents a promising avenue for preventive strategies and personalized treatments, including dietary interventions and pharmacological modulation of methylation [39,91]. Collectively, the dynamic nature of DNA methylation in genes like *ESR1*, influenced by diet, chemicals, and/or lifestyle, underscores the potential for reversible epigenetic changes [92]. This opens up new avenues for prevention and intervention through tailored lifestyle modifications and/or drugs that impact the epigenetic landscape. Studying the methylation patterns in breast malignancies could also provide biomarker development for early detection, prognosis, and response to BC therapy.

## 3. Materials and Methods

### 3.1. Analyses of the TCGA BRCA and Breast Cell Line RNA-Seq Datasets

RNA-Seq datasets were downloaded from the UCSC Xena “https://xena.ucsc.edu (accessed on 25 January 2025)” browser and evaluated the clinical characteristics of cancerous and non-cancerous breast tissues [9,43,44]. The site provides gene expression profiles along with mean normalization per gene. These values were generated by UCSC Xena in combining gene expression RNA-Seq data from all TCGA BRCA cohorts (*n* = 1218). The values were mean-centered per gene, and data specific to this cohort were then extracted. Corresponding phenotypic data were also downloaded from the UCSC Xena repository, encompassing information for 1218 patients. Additionally, miRNA mature strand expression by RNA-Seq for 832 patients and Illumina Infinium HumanMethylation450 data for 888 patients were downloaded from “https://xena.ucsc.edu (accessed on 25 January 2025)” [43].

RNA-Seq datasets from a total of 43 cancerous and non-cancerous breast cell lines were downloaded from UCSC Xena “https://ucsc-public-main-xena-hub.s3.us-east-1.amazonaws.com/download/grayBreastCellLines_public%2FgrayBreastCellLineExon_genomicMatrix.gz (accessed on 25 January 2025)” [46,93]. These data contain gene expression profiles for a variety of breast cancer cell lines. In addition, GI50 to 77 therapeutic compounds as well as ER, HER2, and other phenotypic information is available in the TCGA BRCA clinical data.

### 3.2. Generation of Box and Scatter Plots Utilizing TCGA BRCA Data

The Illumina HTSeq FPKM data were downloaded from the UCSC Xena platform [42,43]. Both box and scatter plots were generated to display the distribution of expression levels [9]. The boxes represent the interquartile range (IQR), with whiskers extending to 1.5 times the IQR, and outliers are shown as individual points. Pairwise comparisons were conducted across groups of normal mammary tissue, ER+/PR+ BC, and other BC subtypes using non-parametric tests, such as the Kruskal–Wallis or Mann–Whitney U tests. Adjusted *p*-values (e.g., Bonferroni correction) were annotated on the plots when statistical significance was detected at *p* < 0.05.

### 3.3. Determination of the Spearman’s Rank Correlation Coefficients with TCGA BRCA Data

The Spearman’s rank correlation coefficients were calculated using the cor.test function in R (R: The R Project for Statistical Computing) to evaluate relationships between two gene expression levels. Correlation matrices were generated and their significance was assessed with *p*-values adjusted for multiple comparisons. Correlation values closer to either +1 or −1 were considered as strong positive or negative correlations, respectively.

### 3.4. Development of a Heatmap for Correlation Matrix

Heatmaps were created using the ComplexHeatmap package version 2.16.0 in R to visualize expression patterns across genes, miRNAs, lncRNAs, and *ESR1*. Hierarchical clustering was applied to samples, with distances calculated using the Spearman correlation coefficients. Log-transformed CPM values were used for data normalization, and color gradients reflected the intensity of expression profiles. A correlation heatmap was generated and analyzed for the enrichment of various genomic factors.

### 3.5. Statistical Analysis

The R software version 4.3.3 “https://www.r-project.org (accessed on 25 January 2025)” was used to perform descriptive statistical analyses, including mean, standard deviation, and fold changes on relevant gene expression levels across diverse groups. Statistical comparisons in *p*-values adjusted for multiple testing using the FDR (False Discovery rate) method.

## 4. Conclusions

It is unequivocal that *ESR1* plays a crucial role in promoting the growth and maintenance of BCs, especially the most prevalent hormone-sensitive BC category [6,8]. The interactions between *ESR1* and certain lncRNAs and/or miRNAs can contribute to endocrine therapy resistance, making it a critical target for breast pathogenesis and its potential therapeutics [89,94]. These scenarios are exemplified and verified by both publicly available TCGA BRCA and 43 breast cell line RNA-Seq datasets [42,46]. Specifically, MEG3 could play a tumor-suppressive role, resulting in a promising therapeutic target for modulating disease pathways involved in ER+/PR+ BC. MiR-30a likely subsidizes cellular homeostasis by suppressing oncogenic drivers such as *ESR1* and ratifying the apoptotic pathway. The positive correlations of miR-30a and MLL with *ESR1* indicate their key roles in chromatin remodeling and in the activation of tumor suppression. Additionally, MEG3 can serve as a modulator in therapies that target PARP inhibitors [95]. The negative correlations of MEG3 and miR-30a with *ESR1* underscore their potential roles in reducing estrogen-driven proliferation for the management of BCs. These inverse relationships suggest therapeutic opportunities that could modulate *ESR1* levels through non-coding RNAs like MEG3 and miR-30a. However, there is a complex interplay between miRNAs and lncRNAs that may result in the suppression of *ESR1*-regulated genes. Overall, the dynamic nature of DNA methylation in genes like *ESR1* provides new insights into BC prevention and/or intervention through tailored lifestyle modifications [92,96]. Based on the above considerations, we conclude that the molecular events involved in *ESR1*-mediated activation of estrogen signaling are influenced by concerted interactions of a number of miRNAs and/or lncRNAs, which helps enlighten on an improved understanding of breast pathogenesis and its therapeutic interventions. Additional studies involving human primary breast tumor tissues and/or pertinent cell lines provide a detailed understanding of the regulation of *ESR1* and its correlation to specific lncRNAs and miRNAs for more targeted therapies for BCs, especially for the prevention and/or treatment of the most prevalent hormone-dependent subtype.

This study has certain limitations, even though it identifies strong correlations between *ESR1* and specific lncRNAs, miRNAs, and DNA methylation sites, utilizing both TCGA BRCA and breast cell line RNA-Seq datasets, and supports the conclusions. While these correlations are significant, TCGA BRCA samples involve heterogeneity, including pathological stages, ages, demographics, and numbers in cancerous and non-cancerous tissues; thus, data interpretation should be made carefully. Further experimental studies are necessary to reveal if these molecules analyzed directly impact *ESR1* expression and whether they are part of broader regulatory networks. However, the study’s cross-sectional design limits the ability to infer temporal relationships with lncRNAs, miRNAs, and *ESR1* expression. Therefore, analyses with more cancerous and non-cancerous breast samples involving demographic information and advanced technological settings would be beneficial for specific conclusions.

## Figures and Tables

**Table 1:** Determination of correlation coefficients between DNA methylation and *ESR1* expression using TCGA BRCA RNA-Seq datasets.

CpG	Correlation	Raw *p* Value	Adjusted *p* Value
cg23009221	−0.43721	6.73 × 10^−23^	3.63 × 10^−21^
cg00601836	−0.65592	6.44 × 10^−58^	3.48 × 10^−56^
cg19411146	−0.44922	3.15 × 10^−24^	1.70 × 10^−22^
cg09646983	−0.49705	4.46 × 10^−30^	2.41 × 10^−28^
cg03037684	0.086458	0.063919	1.000
cg07584093	−0.52222	4.67 × 10^−33^	2.52 × 10^−31^
cg17706972	−0.45091	2.03 × 10^−24^	1.10 × 10^−22^
cg17264271	−0.39943	4.77 × 10^−19^	2.58 × 10^−17^
cg07671949	−0.48692	9.27 × 10^−29^	5.00 × 10^−27^
cg20627916	−0.44493	9.55 × 10^−24^	5.16 × 10^−22^
cg21157690	−0.3816	2.15 × 10^−17^	1.16 × 10^−15^
cg23164938	−0.33446	1.74 × 10^−13^	9.42 × 10^−12^
cg15543523	−0.67757	4.20 × 10^−63^	2.27 × 10^−61^
cg15980539	−0.3692	2.66 × 10^−16^	1.44 × 10^−14^
cg07455133	−0.2602	1.49 × 10^−8^	8.02 × 10^−7^
cg25490334	0.210539	5.25 × 10^−6^	0.000284
cg01715172	−0.58812	3.84 × 10^−44^	2.07 × 10^−42^
cg03732055	−0.28882	2.74 × 10^−10^	1.48 × 10^−8^
cg12209876	−0.00373	0.936474	1.000
cg20893956	−0.33864	8.34 × 10^−14^	4.50 × 10^−12^
cg00920970	−0.36435	6.90 × 10^−16^	3.72 × 10^−14^
cg04211581	−0.40391	1.77 × 10^−19^	9.55 × 10^−18^
cg24900983	−0.48253	3.35 × 10^−28^	1.81 × 10^−26^
cg07059469	0.004822	0.917859	1.000
cg08884395	−0.61707	1.30 × 10^−49^	7.00 × 10^−48^
cg21608605	−0.62193	1.37 × 10^−50^	7.40 × 10^−49^
cg07619683	−0.44714	1.49 × 10^−23^	8.05 × 10^−22^
cg21614759	−0.42071	3.72 × 10^−21^	2.01 × 10^−19^
cg08907436	−0.44323	1.47 × 10^−23^	7.96 × 10^−22^
cg04063345	−0.47197	6.79 × 10^−27^	3.67 × 10^−25^
cg18007957	−0.4478	4.56 × 10^−24^	2.46 × 10^−22^
cg23467008	−0.21278	4.14 × 10^−6^	0.000224
cg21265702	−0.6346	3.25 × 10^−53^	1.75 × 10^−51^
cg01321962	−0.53427	2.60 × 10^−35^	1.40 × 10^−33^
cg11813455	−0.42858	5.63 × 10^−22^	3.04 × 10^−20^
cg26089753	−0.66545	3.80 × 10^−60^	2.05 × 10^−58^
cg23165623	−0.48253	3.35 × 10^−28^	1.81 × 10^−26^
cg09414638	−0.15151	0.001116	0.060274
cg22839866	−0.20121	1.37 × 10^−5^	0.000741
cg07746998	−0.33584	1.37 × 10^−13^	7.39 × 10^−12^
cg02285263	−0.38209	1.94 × 10^−17^	1.05 × 10^−15^
cg02720618	−0.39546	1.14 × 10^−18^	6.15 × 10^−17^
cg10441070	−0.53198	5.70 × 10^−35^	3.08 × 10^−33^
cg27316393	−0.42427	1.59 × 10^−21^	8.60 × 10^−20^
cg24764793	−0.42745	7.40 × 10^−22^	4.00 × 10^−20^
cg20253551	−0.38795	5.68 × 10^−18^	3.07 × 10^−16^
cg11251858	−0.38646	7.80 × 10^−18^	4.21 × 10^−16^
cg15626350	−0.49488	8.64 × 10^−30^	4.67 × 10^−28^
cg07189962	−0.50757	1.72 × 10^−31^	9.30 × 10^−30^
cg00655307	−0.42447	1.52 × 10^−21^	8.19 × 10^−20^
cg10939667	−0.62799	7.89 × 10^−52^	4.26 × 10^−50^
cg05171584	−0.43805	5.45 × 10^−23^	2.94 × 10^−21^
cg21950534	−0.43511	1.14 × 10^−22^	6.14 × 10^−21^
cg01777019	−0.22291	1.37 × 10^−6^	7.42 × 10^−5^

## Data Availability

Data reported in this study are included in this manuscript. The datasets analyzed here are available from the corresponding authors upon reasonable request.

## Supplementary Materials

The following supporting information can be downloaded at https://www.mdpi.com/article/10.3390/ijms26073101/s1.

## Abbreviations

BC	Breast cancer
ER	Estrogen receptor
PR	Progesterone receptor
HER2	Human epidermal growth factor receptor 2
*ESR1*	Estrogen receptor 1
lncRNA	long non-coding RNA
miRNA	Micro RNA
TNBC	Triple-negative breast cancer
BRCA	Breast invasive carcinoma
RNA-Seq	RNA sequencing
FAS	Fatty acid synthetase
TCGA	The cancer genomic atlas
