# Supplementary material for: Harnessing the Role of ESR1 in Breast Cancer: Correlation with microRNA, lncRNA, and Methylation
Source: Int J Mol Sci. 2025 Mar 27;26(7):3101. doi: 10.3390/ijms26073101 (PMC11988918; doi:10.3390/ijms26073101)

Supplementary Materials: Figure S1: ESR1 DNA methylation and gene expression. Colors used were: normal tissue – red; ER+/PR+ samples – green; other BC subtypes – blue.

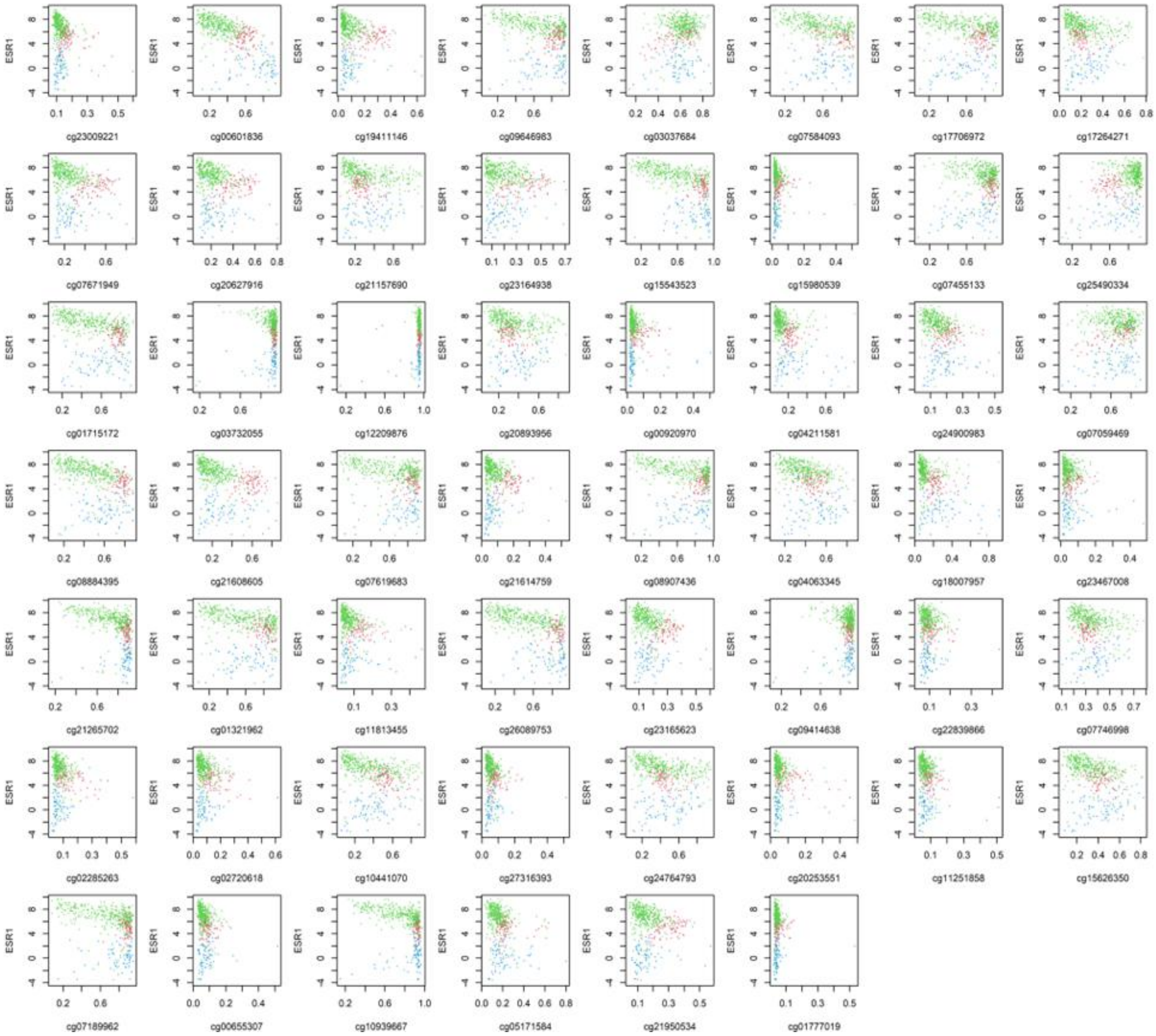

Supplement: Supplementary file 1 [file ijms-26-03101-s001.zip › ijms-3482242-supplementary.pdf]
